# Structural bases of TRP channel TRPV6 allosteric modulation by 2-APB

**DOI:** 10.1038/s41467-018-04828-y

**Published:** 2018-06-25

**Authors:** Appu K. Singh, Kei Saotome, Luke L. McGoldrick, Alexander I. Sobolevsky

**Affiliations:** 1 Department of Biochemistry and Molecular Biophysics, Columbia University, 650 West 168th Street, New York, NY 10032 USA; 2 Integrated Program in Cellular, Molecular and Biomedical Studies, Columbia University, 650 West 168th Street, New York, NY 10032 USA

## Abstract

Transient receptor potential (TRP) channels are involved in various physiological processes, including sensory transduction. The TRP channel TRPV6 mediates calcium uptake in epithelia and its expression is dramatically increased in numerous types of cancer. TRPV6 inhibitors suppress tumor growth, but the molecular mechanism of inhibition remains unknown. Here, we present crystal and cryo-EM structures of human and rat TRPV6 bound to 2-aminoethoxydiphenyl borate (2-APB), a TRPV6 inhibitor and modulator of numerous TRP channels. 2-APB binds to TRPV6 in a pocket formed by the cytoplasmic half of the S1–S4 transmembrane helix bundle. Comparing human wild-type and high-affinity mutant Y467A structures, we show that 2-APB induces TRPV6 channel closure by modulating protein–lipid interactions. Mutagenesis and functional analyses suggest that the identified 2-APB binding site might be present in other members of vanilloid subfamily TRP channels. Our findings reveal a mechanism of ion channel allosteric modulation that can be exploited for therapeutic design.

## Abstract (web summary)

The transient receptor potential channel TRPV6 mediates calcium uptake in epithelia and its expression is increased in several cancer types. Here, authors present structures of TRPV6 bound to 2-APB, a TRPV6 inhibitor, and show that 2-APB induces TRPV6 channel closure by modulating protein–lipid interactions.

## Introduction

The transient receptor potential (TRP) ion channel superfamily comprises members that are involved in various physiological functions ranging from sensory transduction to calcium homeostasis^1^. Aberrant regulation of TRP channels results in various diseases^2^, including numerous types of cancer. TRPV6, a highly calcium-selective TRP channel, is overexpressed in endometrial cancers, leukemia, and carcinomas of the breast, prostate, colon, ovarian, and thyroid^3–18^. TRPV6 has been implicated in tumor development and progression, and its overexpression pattern correlates with the aggressiveness of the disease^4,8,9,15,19–21^. Ca^2+^ is a critical regulator of cell proliferation, suggesting a role for TRPV6 in the potentiation of calcium-dependent cell proliferation and inhibition of apoptosis^17^. Inhibitors of TRPV6 may, therefore, offer a novel therapeutic strategy for treatment of TRPV6-rich tumors^12,17,20,22^. A limited number of small-molecule^23–26^ and peptide^27^ inhibitors of TRPV6 have been identified as potential leads for cancer treatment, but advances in drug development are hampered by lack of knowledge about the possible molecular mechanisms of TRPV6 inhibition.

A membrane-permeable compound 2-aminoethoxydiphenyl borate (2-APB), one of the few known small-molecule inhibitors of TRPV6, has been shown to attenuate tumor growth and invasiveness in human cancer cell lines in vitro^26^. 2-APB was initially characterized as an inhibitor of Ins(1,4,5)P3 receptor-induced Ca^2+^ release^28^, but was later shown to modulate the functions of different ion channels, including calcium release-activated^29^ and two-pore potassium^30^ channels. 2-APB modulation of TRP channels^31^, includes activation of TRPV1, TRPV2, TRPV3, TRPA1, and TRPM6^32–35^ and inhibition of TRPM2, TRPM7, TRPC3, TRPC6, and TRPC7^36–38^. The promiscuousness of 2-APB makes it an important research tool to characterize physiological function and biophysical properties of ion channels. However, the potential of 2-APB as a lead compound for drug design remains limited because its mechanisms of action remain poorly understood. To address this knowledge gap, we embarked on structural studies of TRPV6 inhibition by 2-APB.

Here, we solve cryo-EM and crystal structures of human and rat TRPV6 in complex with 2-APB, which binds in a pocket formed by the cytoplasmic half of the S1–S4 transmembrane helix bundle. By comparing our structures, we find that 2-APB induces TRPV6 channel closure by modulating protein–lipid interactions. Mutagenesis and functional analyses suggest that the 2-APB binding site is likely present in other members of the vanilloid subfamily of TRP channels. Our proposed mechanism of TRPV6 inhibition by 2-APB, therefore, contributes to the general principles of TRP channel regulation by small hydrophobic molecules.

## Results

### Crystal structure of rat TRPV6 in complex with 2-APB

**Fig. 1:**
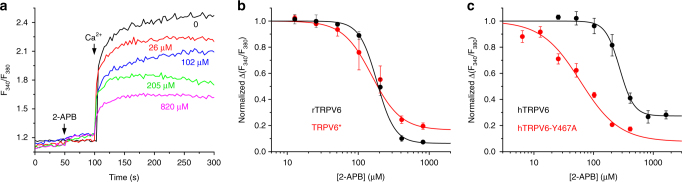
2-APB inhibition of Ca^2+^ uptake through wild-type and mutant TRPV6 channels. **a** Ratiometric Fura-2 fluorescence curves recorded from HEK 293 cells expressing TRPV6* in response to application of 2 mM Ca^2+^ after preincubation of cells in different concentrations of 2-APB. Arrows indicate the times when 2-APB and Ca^2+^ were added. These experiments were repeated independently three times with similar results. **b** Dose–response curves for 2-APB inhibition of Ca^2+^ uptake calculated for wild-type rTRPV6 (black) and TRPV6* (red) (*n* = 3 for all measurements). The changes in the fluorescence intensity ratio at 340 and 380 nm (*F*_340_/*F*_380_) evoked by addition of 2 mM Ca^2+^ after preincubation with various concentrations of 2-APB were normalized to the maximal change in *F*_340_/*F*_380_ after addition of 2 mM Ca^2+^ in the absence of 2-APB. Straight lines through the data points are fits with the logistic equation, with the mean ± SEM values of the half-maximum inhibitory concentration (IC_50_), 184 ± 8 and 156 ± 20 μM, and the maximal inhibition, 93.6 ± 1.1% and 83.5 ± 2.9%, for rTRPV6 and TRPV6*, respectively. **c** Dose–response curves for 2-APB inhibition of Ca^2+^ uptake calculated for wild-type hTRPV6 (black) and hTRPV6-Y467A (red) (*n* = 3 for all measurements) are fits with the logistic equation, with the parameter values IC_50_ = 274 ± 27 and 60 ± 13 μM, and the maximal inhibition, 72.6 ± 2.7% and 92.4 ± 8.1%, for hTRPV6 and hTRPV6-Y467A, respectively. The leftward shift of the 2-APB dose–response curve of hTRPV6-Y467A, when compared to the dose–response curve of wild-type hTRPV6, indicates an increased affinity of the channel for 2-APB. Error bars represent SEMs

**Fig. 2:**
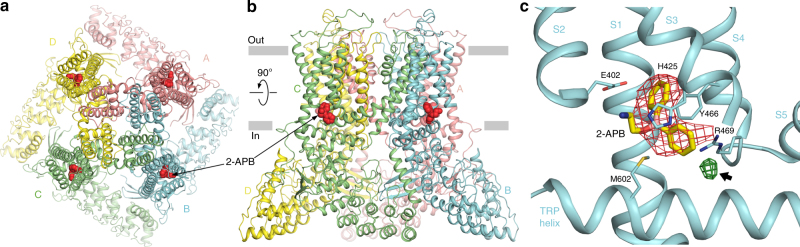
Crystal structure of rat TRPV6 in complex with 2-APB. **a**, **b** Top (**a**) and side (**b**) views of TRPV6* in complex with 2-APB. The molecules of 2-APB are shown as red space-filling models. Four TRPV6* subunits are colored pink, cyan, green, and yellow. **c** Close-up view of the 2-APB binding site with 2-APB (yellow) and surrounding residues shown as sticks. Red mesh shows positive electron density for 2-APB in the Fo–Fc omit map contoured at 3*σ*. Green mesh indicated by the arrow shows electron density for the brominated derivative of 2-APB (2-APB-Br) in the anomalous difference Fourier map contoured at 3*σ*

We used the TRPV6* construct that we developed earlier by modifying rat TRPV6 (rTRPV6) for crystallization (see Methods). TRPV6* exhibits Ca^2+^ permeability and Gd^3+^ block similar to the wild-type channel^39^. Importantly, in fluorimetric assays 2-APB inhibited calcium uptake through TRPV6* (Fig. 1a, b) with a similar efficacy (IC_50_ = 156 ± 20 μM, *n* = 3) as in wild-type channels (IC_50_ = 184 ± 8 μM, *n* = 4). We solved the crystal structure of TRPV6* in complex with 2-APB (TRPV6*_2-APB_) by molecular replacement using the ligand-free TRPV6* structure^39^ as a search model (Supplementary Table 1). The TRPV6*_2-APB_ structure (Fig. 2a, b) does not exhibit substantial conformational rearrangements when compared to TRPV6* (the root mean square deviation, RMSD = 0.505). TRPV6 has four subunits that form a transmembrane domain (TMD) with a central ion channel pore, an intracellular skirt domain enclosing a large cavity, and amphipathic TRP helices that run nearly parallel to the membrane and interact with both the TMD and the skirt. The TMD is composed of transmembrane helices S1–S6 and a re-entrant pore loop (P-loop) between S5 and S6. The first four transmembrane helices form a bundle to constitute the S1–S4 domain. The domain homologous to the S1–S4 domain acts as the voltage sensor in voltage-gated channels^40^, but its role in allosteric gating of TRP channels remains obscure. The pore domain of each subunit includes, S5, the P-loop, and S6, and is packed against the S1–S4 domain of the neighboring subunit in a domain-swapped arrangement^39,41^.

### 2-APB binding pocket in rat TRPV6

**Fig. 3:**
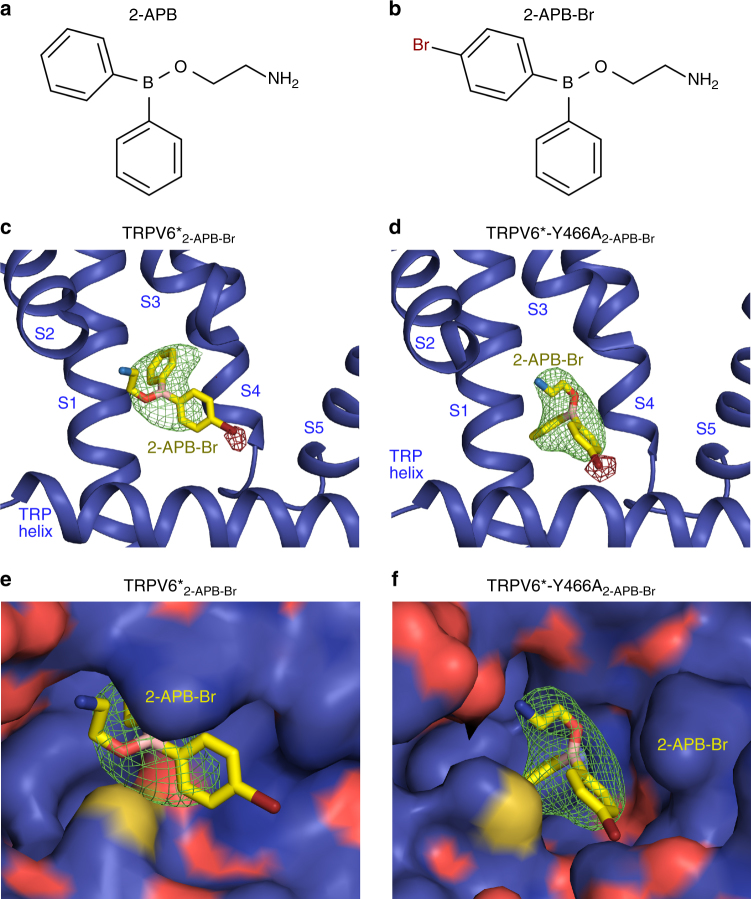
Binding of brominated 2-APB to TRPV6* and TRPV6*-Y466A. **a**, **b** Chemical structures of 2-APB (**a**) and 2-APB-Br (**b**). **c**, **d** Close-up view of the 2-APB binding site in TRPV6*_2-APB-Br_ (**c**) and TRPV6*-Y466A_2-APB-Br_ (**d**). The molecule of 2-APB-Br is in stick representation, with the Fo–Fc omit map (2.5*σ*) shown as a green mesh and the anomalous difference Fourier maps at 3*σ* for TRPV6*_2-APB-Br_ and 5.5*σ* for TRPV6*-Y466A_2-APB-Br_ shown as a brown mesh. **e**, **f** Surface representation of the 2-APB binding pocket in TRPV6*_2-APB-Br_ (**e**) and TRPV6*-Y466A_2-APB-Br_ (**f**). Note the different orientations (poses) of the 2-APB-Br molecule in the two structures

In each subunit of the TRPV6*_2-APB_ tetramer, a pocket formed by the intracellular portions of the S1–S4 helices and the membrane-facing sides of the amphipathic TRP helices contained a strong density that matches the size and shape of 2-APB and was not observed in the TRPV6* structure (Fig. 2c, Supplementary Figure 1a, b). To verify this 2-APB binding site crystallographically, we used a heavy atom bromine derivative of 2-APB (2-APB-Br; Fig. 3a, b) and collected diffraction data at a wavelength of 0.92 Å from crystals of TRPV6* grown in the presence of 2-APB-Br (Supplementary Table 1). These data revealed an anomalous difference Br peak at the putative 2-APB electron density and unambiguously identified the orientation of 2-APB in the binding pocket (Fig. 2c, Fig. 3). Notably, this binding pocket is distinct from the pocket at the S4–S5 interface that binds an activating lipid in TRPV6^41^, agonists, such as resiniferatoxin (RTX) and capsaicin, and antagonists, such as capsazepine (CPZ), in TRPV1^42^, and the inhibitor econazole in TRPV5^43^. However, the 2-APB binding site in TRPV6* overlaps with a lipid binding site found in TRPV1^42^, TRPV2^44,45^, TRPV5^43^, and TRPV6^41^ (Supplementary Figure 1c–f); a Ca^2+^ binding site in TRPM4^46^; putative binding sites for phosphoinositides in TRPA1^47^; and menthol in TRPM8^48^, suggesting an important role for this site in modulating TRP channel function.

2-APB binding is apparently mediated by multiple interactions (Fig. 2c). E402 in S2 appears to interact with the amino group of the 2-APB tail. In addition, the two phenyl rings of 2-APB are surrounded by the imidazole group of H425 in S3, the guanidinium group of R469 in S4, the hydrophobic side chains of Y466 in the S4–S5 linker, and M602 in the TRP domain.

### Binding Site Mutations Increase 2-APB Modulation Potency

**Fig. 4:**
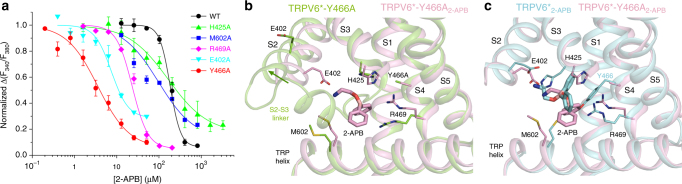
Probing of 2-APB binding site in TRPV6 by mutagenesis. **a** Dose–response curves for 2-APB inhibition of wild-type and mutant rat TRPV6 channels with residues in the 2-APB binding pocket individually substituted to alanine. Error bars represent SEMs (*n* = 3–7). **b** Close-up view of superposition of the 2-APB binding pocket in the TRPV6*-Y466A (green) and TRPV6*-Y466A_2-APB_ (pink) crystal structures with the 2-APB molecule and surrounding residues shown as sticks. Green arrow indicates displacement of S2 in the absence of 2-APB, accompanied by increased order in the S2–S3 linker. **c** Close-up view of superposition of the 2-APB binding pocket in the TRPV6*_2-APB_ (cyan) and TRPV6*-Y466A_2-APB_ (pink) crystal structures

To further characterize the 2-APB binding site and to examine the contribution of individual residues to 2-APB inhibition, we introduced alanine substitutions at residues in the 2-APB binding pocket (E402, H425, Y466, R469, and M602) and tested 2-APB inhibition. In stark contrast to our expectations, the mutations did not weaken the inhibitory potency of 2-APB, but either had little effect (IC_50_ = 165 ± 32 μM, *n* = 3 for H425A; IC_50_ = 98 ± 19 μM, *n* = 3 for M602A), or resulted in a remarkable 8- to 50-fold increase in 2-APB potency (IC_50_ = 7.5 ± 2.1 μM, *n* = 3 for E402A; IC_50_ = 3.62 ± 0.54 μM, *n* = 4 for Y466A; IC_50_ = 23.4 ± 1.6 μM, *n* = 4 for R469A) compared to wild-type channels (Fig. 4a).

The strongest increase in potency of calcium uptake inhibition by 2-APB was observed for the mutant TRPV6*-Y466A. To shed light on the structural basis of this phenotype, we crystallized TRPV6*-Y466A in the presence and absence of 2-APB (Fig. 4b, Supplementary Table 1). In the presence of 2-APB, a strong density with the characteristic shape of 2-APB is present in the S1–S4 binding pocket, similar to TRPV6*_2-APB_. Interestingly, both the shape of the 2-APB density in the Fo–Fc map of TRPV6*-Y466A_2-APB_ and the strong Br anomalous peak for TRPV6*-Y466A co-crystallized with 2-APB-Br (Fig. 3) indicate that the binding pose for 2-APB in TRPV6*-Y466A_2-APB_ is different from its pose in TRPV6*_2-APB_. In the absence of 2-APB, the C-terminal (cytoplasmic) part of S2 in TRPV6*-Y466A is splayed away from the binding pocket by ~4 Å relative to its position in TRPV6*-Y466A_2-APB_ (Fig. 4b). In turn, 2-APB seems to glue the S2 helix back, perhaps by virtue of its tail amino group interaction with E402 (Fig. 4c).

**Fig. 5:**
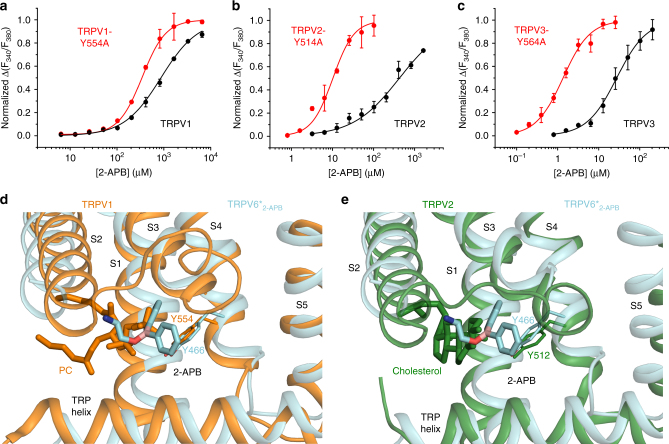
Probing of the 2-APB binding site in different TRPV channels. **a**–**c** Dose–response curves for 2-APB activation of wild-type (black) and mutant (red) rat TRPV1 (**a**), rat TRPV2 (**b**), and mouse TRPV3 (**c**) channels with alanine substitution of the conserved tyrosine in the presumed 2-APB binding pocket. Each data point represents an average of *n* = 3–7 measurements with error bars representing SEMs. **d**, **e** Close-up view of superposition of the 2-APB binding pocket in the TRPV6*_2-APB_ crystal structure (cyan) and the corresponding regions of rat TRPV1 (**d**) PDB ID: 5IRX, orange and rabbit TRPV2 (**e**) PDB ID: 5AN8, green. Phosphatidylcholine (PC), cholesterol and 2-APB molecules as well as the side chain of the conserved tyrosine are shown as sticks

Y466 in TRPV6* is highly conserved throughout the entire TRPV subfamily (Supplementary Figure 2). We wondered whether mutating the homologous tyrosine in TRPV1, TRPV2, and TRPV3 would affect the potency of 2-APB as an agonist^32^ for these channels. Indeed, for all three, we observed leftward shifts of their 2-APB concentration dependencies (Fig. 5a–c), indicating 3-, 40-, and 20-fold increases in 2-APB agonist potency for TRPV1, TRPV2, and TRPV3, respectively. In available structures of TRPV1^42^ and TRPV2^44,45^, the tyrosine side chain projects into the 2-APB binding pocket in a fashion similar to Y466 in TRPV6* (Fig. 5d, e), suggesting its conserved structural role in 2-APB-dependent modulation. On the other hand, the lack of measurable 2-APB modulation of TRPV4 and TRPV5^32^ despite high sequence conservation (Supplementary Figure 2), suggests that there are other determinants of modulation of TRPV channels by 2-APB.

The increased potency of 2-APB modulation observed in inhibited (TRPV6) and activated (TRPV1–3) channels caused by the conserved tyrosine substitution in S4 supports the idea that these channels share a common site for 2-APB binding. We hypothesize that the increase, rather than reduction of 2-APB potency caused by mutations in its binding pocket is rooted in competition between 2-APB and the S1–S4 lipid (lipid 1, see the next section) that occupies this site in the absence of 2-APB (Fig. 5d, e, Supplementary Figure 1). Therefore, the lack of 2-APB modulation of TRPV4 and TRPV5^32^ might be due to tighter binding of the S1–S4 lipid and inability of 2-APB to displace it. In turn, a more loose binding pocket, such as the one observed in TRPV6*-Y466A (Fig. 4b), might cause reduction in TRPV6* affinity to the S1–S4 lipid and, as a result, increased affinity to 2-APB. We decided to further explore the structural mechanism of 2-APB inhibition by comparing TRPV6 structures in the open and 2-APB-inhibited closed states.

### 2-APB-Induced Closure of Human TRPV6

**Fig. 6:**
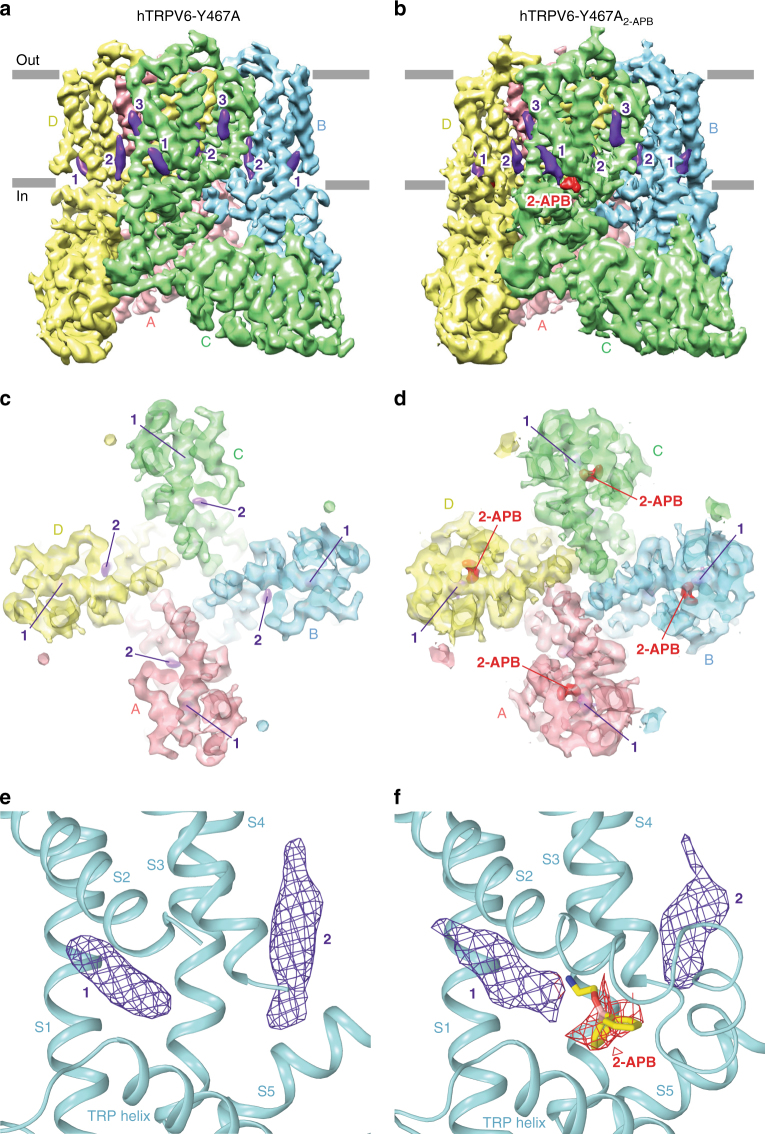
Open and 2-APB-bound closed cryo-EM structures of human TRPV6. **a**, **b** 3D reconstructions of hTRPV6-Y467A (**a**) and hTRPV6-Y467A_2-APB_(**b**) viewed parallel to the membrane, with cryo-EM density for hTRPV6 subunits colored pink, cyan, green, and yellow; lipid in purple and 2-APB in red. **c**, **d** Semi-transparent cryo-EM density for the ion channel in open hTRPV6-Y467A (**c**) and closed hTRPV6-Y467A_2-APB_ (**d**) viewed intracellularly. **e**, **f** Close-up views of the binding pocket of 2-APB (yellow) in hTRPV6-Y467A (**e**) and hTRPV6-Y467A_2-APB_ (**f**). Red and purple mesh shows cryo-EM density for 2-APB and lipids, respectively

**Fig. 7:**
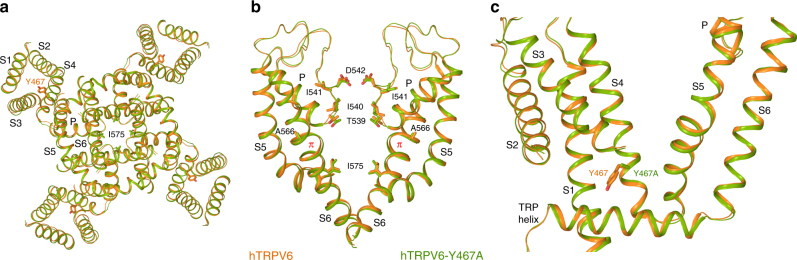
Comparison of hTRPV6 and hTRPV6-Y467A cryo-EM structures. **a**–**c** Superimposed are the transmembrane domain viewed intracellularly (**a**) and the pore-forming region with the front and back subunits omitted for clarity (**b**), and the transmembrane domain of a single subunit (**c**) viewed parallel to the membrane for the cryo-EM structures of hTRPV6 (orange, PDB ID: 6BO8) and hTRPV6-Y467A (green). Residues contributing to the selectivity filter and gating as well as Y467 and Y467A are shown as sticks. The local *π* helical conformations of S6 associated with the open channel are indicated (red labels)

We have only been able to crystallize rat TRPV6 in the closed state, precluding a clear view of conformational changes associated with 2-APB-dependent inhibition. We thus turned to human TRPV6 (hTRPV6), of which we recently resolved the open-state structure by cryo-electron microscopy (cryo-EM)^41^. Unfortunately, our attempts to determine the structure of wild-type hTRPV6 in complex with 2-APB were not successful, likely due to low-affinity binding (IC_50_ = 274 ± 27 μM, *n* = 3, Fig. 1c). In order to increase the affinity of hTRPV6 to 2-APB, we introduced the Y467A mutation, homologous to Y466A in TRPV6*. As expected, hTRPV6-Y467A showed 4.5-fold increased potency of 2-APB inhibition (IC_50_ = 60 ± 13 μM, *n* = 3) compared to hTRPV6 (Fig. 1c). We determined the cryo-EM structure of apo hTRPV6-Y467A (Supplementary Table 2), which is in the open-state conformation (Fig. 6a, c, e and Supplementary Figure 3a, c, e, g), and it is nearly identical (RMSD = 0.765) to the cryo-EM structure of wild-type hTRPV6 (Fig. 7). Lipids are observed in positions similar to those seen in hTRPV6^41^; lipid 1 is bound in a pocket formed by the intracellular half of the S1–S4 domains and C-terminal part of TRP helix, while lipid 2 is above the S4–S5 linker and contacts both S3 and S4 of the same subunit, and S5–S6 of the neighboring subunit. We then solved the cryo-EM structure of hTRPV6-Y467A in complex with 2-APB (Fig. 6b, d, f, Supplementary Figure 3b, d, f, h, Supplementary Table 2). Clear density for 2-APB is present in each subunit of hTRPV6-Y467A_2-APB_ tetramer (Fig. 6f), at locations very similar to the binding sites in TRPV6*-Y466A_2-APB_ (Fig. 4b).

**Fig. 8:**
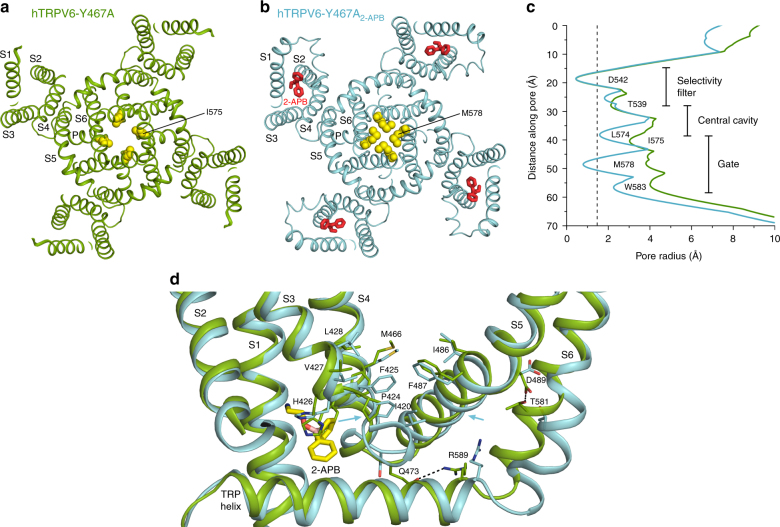
Open and closed pores in cryo-EM structures of hTRPV6-Y467A and hTRPV6-Y467A_2-APB_ and 2-APB-induced conformational changes. **a**, **b** Intracellular view of the transmembrane domain in open-pore hTRPV6-Y467A (**a**) and closed-pore hTRPV6-Y467A_2-APB_ (**b**). Residues forming the narrowest part of the pore in the gate region, I575 in hTRPV6-Y467A (**a**) and M578 in hTRPV6-Y467A_2-APB_ (**b**), are shown as space-filling models (yellow). Molecules of 2-APB are shown as sticks (red). **c** Pore radius calculated using HOLE^66^ for hTRPV6-Y467A (green) and hTRPV6-Y467A_2-APB_ (cyan). **d** Superposition of hTRPV6-Y467A (green) and hTRPV6-Y467A_2-APB_ (cyan), with hydrophobic residues, residues involved in 2-APB binding, residues important for gating, and 2-APB shown as sticks. Blue arrows illustrate movement of the hydrophobic residues to form the hydrophobic cluster. Dashed lines illustrate hydrogen bonds that stabilize the open state

**Fig. 9:**
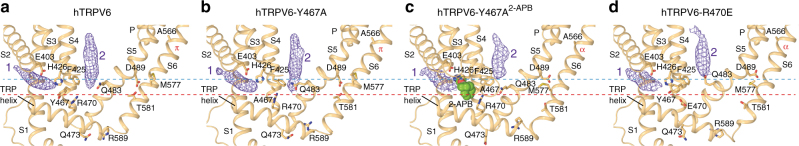
Densities for S1–S4 and activating lipids in cryo-EM structures of hTRPV6. **a**–**d** Close-up views of the transmembrane domain of a single subunit in the cryo-EM structures of hTRPV6 (**a** PDB ID: 6BO8), hTRPV6-Y467A (**b**), hTRPV6-Y467A_2-APB_ (**c**), and hTRPV6-R470E (**d** PDB ID: 6BOA) viewed parallel to the membrane. The molecule of 2-APB is shown as a space-filling model (green). Purple mesh shows cryo-EM density for the S1–S4 (1) and activating (2) lipids, low pass filtered to the same (4.44 Å) resolution. Red and blue dashed lines indicate the lowest (most intracellular) levels reached by the activating lipids in the open (hTRPV6 and hTRPV6-Y467A) and closed (hTRPV6-Y467A_2-APB_ and hTRPV6-R470E) structures, respectively. The local *π* and *α* helical conformations of S6 associated with the open and closed states, respectively, are indicated (red labels)

Most importantly, a pore radius calculation confirmed that channel of hTRPV6-Y467A_2-APB_ is in the closed conformation (Fig. 8), allowing direct structural comparison with the high-resolution open-state structure of hTRPV6 (Fig. 8d). 2-APB binding is accompanied by movement of F425 and P424 in S3 toward the S4–S5 linker. As a result, hydrophobic residues from the S2–S3 linker (I420), N-terminal portion of S3 (P424, F425, V427, and L428), C-terminal portion of S4 (M466), and S4–S5 linker (I486 and F487) come closer together to form a hydrophobic cluster at the bottom of the binding site for the activating lipid (lipid 2). In both hTRPV6^41^ and hTRPV6-Y467A, the activating lipid head penetrates this area to interact with Q483 and R470 (Fig. 9a, b). In the hTRPV6-Y467A_2-APB_ structure, the activating lipid head is extruded by the hydrophobic cluster, and is correspondingly shifted extracellularly. This activating lipid movement is similar to that observed in hTRPV6-R470E^41^, where the R470E mutation switched hTRPV6 from the open to closed state (Fig. 9c, d).

## Discussion

**Fig. 10:**
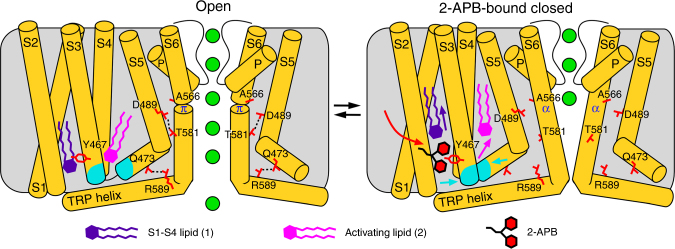
Mechanism of TRPV6 inhibition by 2-APB. Cartoons represent the structural changes that start with binding (red arrow) of 2-APB, which displaces the S1–S4 lipid (lipid 1, purple) and promotes formation of the hydrophobic cluster (cyan). Formation of the cluster displaces the activating lipid (lipid 2, pink) and eliminates hydrogen bonds (dashed lines), which stabilize the open state by energetically compensating the unfavorable *α*-to-*π* helical transition in S6. As S6 turns *α*-helical, the channel closes and its pore becomes impermeable to ions (green spheres)

Comparing the open-state structures of hTRPV6 and the 2-APB-bound closed-state structures, we conclude that 2-APB allosterically modulates TRPV6 channel conformation through its perturbation of lipid–protein interactions (Fig. 10, Supplementary Movie 1). Competing for the same binding site (e.g., see Supplementary Figure 1f), 2-APB wrings out the S1–S4 lipid. This promotes the formation of a hydrophobic cluster that squeezes out the activating lipid relative to its binding locus in the open state structure of TRPV6^41^. The accompanying rearrangements of the transmembrane helices eliminate hydrogen bonds between Q473 in the S4–S5 linker and R589 in the TRP helix, and between D489 in the S5 helix and T581 in the S6 helix. These bonds compensate for the unfavorable *α*-to-*π* helical transition in S6 that occurs during channel opening and their removal makes S6 *α*-helical and promotes channel closure.

We hypothesize that 2-APB similarly modulates the activity of other TRPV channels by manipulating their interactions with bound lipids, but that the functional outcome (activation, inhibition, or no effect) depends on the exact nature of the channel–lipid complex and whether the lipids have a positive or negative regulatory role in channel gating. The 2-APB binding site identified in the present study can, therefore, be exploited for the design of small hydrophobic molecules that can alter channel function in a specific way, possibly leading to novel therapeutic strategies.

## Methods

### Construct

For crystallization experiments, we used the previously developed construct TRPV6*^39^, which includes wild-type rat TRPV6 (rTRPV6) residues 1–668 and three point mutations (I62Y, L92N, and M96Q), and the TRPV6*-Y466A construct, which contains an additional point mutation, Y466A. For calcium uptake measurement experiments, we used TRPV6*, rTRPV6, and rTRPV6 with individual alanine substitutions (rTRPV6-E402A, rTRPV6-H425A, rTRPV6-Y466A, rTRPV6-R469A, and rTRPV6-M602A), wild-type human TRPV6 (hTRPV6), hTRPV6 with a single tyrosine-to-alanine substitution (hTRPV6-Y467A), wild-type rat TRPV1 (rTRPV1), rat TRPV2 (rTRPV2) and mouse TRPV3 (mTRPV3), and their mutant versions with single tyrosine-to-alanine substitutions (rTRPV1-Y554A, rTRPV2-Y514A, and mTRPV3-Y564A, respectively). For cryo-EM experiments, we used hTRPV6 and hTRPV6-Y467A constructs.

### Expression and Purification

The TRPV6*, TRPV6*-Y466A, and hTRPV6-Y467A constructs were expressed and purified as previously described for TRPV6_cryst_^49^ and hTRPV6^41^ with slight modification. Briefly, for our crystallographic studies, TRPV6* was introduced into a pEG BacMam vector^50^ with a C-terminal thrombin cleavage site (LVPRG) followed by eGFP and a streptavidin affinity tag (WSHPQFEK) for expression in baculovirus-transduced, suspension-adapted HEK 293 GnTI^−^ cells (ATCC, Cat#CRL-3022). For our cryo-EM studies, hTRPV6-Y467A was introduced into a similar pEG BacMam vector, but without GFP. In total, 48–72 h after transduction with baculovirus virus, HEK 293 GnTI^−^ cells were harvested, washed with PBS pH 8.0, and after sonication, cellular membranes were prepared. The membranes were then mechanically homogenized, and solubilized for 1 h in 150 mM NaCl, 20 mM Tris-HCl pH 8.0, 1% DDM (n-dodecyl-β-d-maltopyranoside), 0.1% cholesteryl hemisuccinate (CHS), and 1 mM β-mercaptoethanol (BME) for hTRPV6-Y467A. CHS was omitted while purifying TRPV6* and TRPV6*-Y466A. After solubilizing the membranes, any remaining insoluble material was pelleted by ultracentrifugation (186,000×*g*, 40 min) and the soluble fraction was incubated with Streptavidin-linked resin for 10–14 h, rotating at 4 °C. The resin was washed with 10 column volumes of 150 mM NaCl, 20 mM Tris-HCl pH 8.0, 1 mM BME, 0.1% DDM, and 0.01% CHS for hTRPV6, and without CHS for TRPV6* and TRPV6*-Y466A. The protein was eluted in the same buffer supplemented with 2.5 mM d-desthiobiotin, and was further purified by size exclusion chromatography. The tetrameric peak fractions were pooled and for hTRPV6-Y467A, the protein was reconstituted in amphipols and concentrated to 0.35–0.45 mg/ml for cryo-EM sample preparation. For structural studies using crystallography, TRPV6* and TRPV6*-Y466A were both concentrated to 2.5 mg/ml. For both crystallographic and cryo-EM studies of TRPV6 in complex with 2-APB, 1 mM 2-APB was added to every buffer throughout the purification.

### Crystallization and Structure Determination

Crystals of purified TRPV6* were grown in the hanging drop configuration at 20 °C in the same condition as described previously^49^: it consists of a reservoir solution containing 20–24% PEG 350 MME, 50 mM NaCl, and 50 mM Tris-HCl pH 8.0–8.5. Prior to crystallization, the protein was subjected to ultracentrifugation (Ti100 rotor, 86,500×*g*, 40 min, 4 °C) to remove any aggregated protein. TRPV6* purified in the presence of 1 mM 2-APB produced larger crystals compared to those prepared from purified TRPV6* incubated with the 2-APB for 3 h prior to setting up crystallization trays. The mono-brominated 2-APB (2-APB-Br) was synthesized according to the previously described procedure^51^. Commercially available 1,4-dibromobenzene was mono-lithiated with ^*n*^BuLi and then reacted with commercially available phenylboronic acid pinacol ester to yield borinic acid. Borinic acid was subsequently esterified with 2-aminoethanol to yield the desired 2-APB-Br. A 100-mM stock of 2-APB-Br was prepared in DMSO and added to purified TRPV6* and TRPV6*-Y466A to a final concentration of 1 mM. These mixtures were incubated for 3 h on ice prior to setting up crystallization trays. Crystals were cryo-protected by serial transfer into buffers composed of 100 mM NaCl, 100 mM Tris-HCl pH 8.2, 0.5 mM DDM, and 50 mM ammonium formate, and containing increasing concentrations of PEG 350 MME, with the maximum concentration of 33–36%, and then flash frozen in liquid nitrogen.

X-ray diffraction data collected at APS (beamlines 24-ID-C/E), NSLSII (beamline 17-ID), or ALS (beamline 5.0.2) were indexed, integrated, and scaled using XDS^52^ or HKL2000^53^. The initial phase information and structures were solved by molecular replacement using Phaser^54^ and the structure of rat TRPV6* (PDB ID: 5WO7) as a search probe. Most of TRPV6*, including the ankyrin repeat domain, S1–S4, the pore module and the C-terminal hook, were similar to the TRPV6_cryst_; the rest of the structure was built using the omit map as a guide. The robust electron density for the S4–S5 linker was evident from initial phases obtained by molecular replacement and map features improved further during refinement. The model was refined by alternating cycles of building in COOT^55^ and automatic refinement in Phenix^56^ or Refmac^57^.

### Reconstitution of hTRPV6-Y467A into Amphipols

hTRPV6-Y467A was reconstituted into amphipols as described previously^41^. Briefly, 0.48 mg of hTRPV6-Y467A was incubated with amphipols at 4 °C with constant rotation in a 3:1 amphipols:hTRPV6-Y467A mass ratio. After 3 h, 7–10 mg of Bio-beads SM2 prewet in buffer (20 mM Tris, pH 8.0, 50 mM NaCl) was added to the protein–amphipols mixture. This mixture was then rotated overnight at 4 °C.

### Cryo-EM Sample Preparation and Data Collection

The cryo-EM grids for hTRPV6-Y467A or hTRPV6-Y467A_2-APB_ were prepared as described previously^41^. In short, the gold-coated grids were plasma treated prior to sample application. A Vitrobot Mark IV was used to plunge freeze the grids after the application of 3 µl protein solution with 100% humidity at 5 °C, a blot time of 2 or 3 s, the blot force set to 3, and a wait time of 20 s. A concentration of 0.35 mg/ml was used for the amphipols-solubilized protein.

The hTRPV6-Y467A data were collected on a Tecnai F30 Polara (C_s_ 2.26 mm), operating at 300 kV, equipped with a Gatan K2 Summit electron detection (DED) camera (Gatan, Pleasanton, CA, USA) using Leginon^58^. A total of 3272 micrographs were collected with a pixel size of 0.98 Å across a defocus range of −1.5 to −3.5 µm. The total dose, ~67 e^−^ Å^−2^, was attained by using a dose rate of ~8.0 e^−1^ pixel^−1^ s^−1^ across 40 frames for 8 s total exposure time. The hTRPV6-Y467A_2-APB_ data were collected on a Tecnai F20, operating at 200 kV, equipped with a Gatan K2 Summit electron detection (DED) camera (Gatan, Pleasanton, CA, USA) using Leginon^58^. For hTRPV6-Y467A_2-APB_, a total of 2112 micrographs were collected with a pixel size of 1.22 Å across a defocus range of −1.5 to −3.5 µm. The total dose, ~43 e^−1^ Å^−2^, was attained by using a dose rate of ~8.0 e^−1^ pixel^−1^ s^−1^ across 40 frames for 8 s total exposure time.

### Image Processing

Frame alignment was done using MotionCor2^59^. For both data sets, CTF correction was carried out using Gctf^60^ on nondose-weighted micrographs, while subsequent data processing was done on dose-weighted micrographs. The data processing were performed using Relion 2.1^61^. For both data sets, between 1000 and 1500 particles were manually selected to generate 2D classes that were used as templates for automated particle picking. Five 2D classes were used as references for the automated picking of 642,142 particles from the 3272 hTRPV6-Y466A micrographs. The particle images were binned to a pixel size of 1.96 Å, and screened using 2D classification. The remaining particles were subjected to 3D classification with C1 symmetry using an hTRPV6 cryo-EM structure (EMDB-7120) low pass filtered to 40 Å as a reference. Two additional rounds of 3D classification were performed on 0.98 Å pixel size particle images with C4 symmetry imposed to generate a more homogenous set of 115,126 particles. To generate the final 3D map, we processed this subset of particles using 3D-auto-refine procedure followed by a postprocessing procedure in Relion 2.1.

The hTRPV6-Y467A_2APB_ data set was processed in a similar workflow to that described above and the reported resolutions were estimated using the FSC = 0.143 criterion^62^ on masking-effect-corrected FSC curves calculated between two independent half-maps^63^. The local resolutions were estimated with unfiltered half maps using ResMap^64^ and EM density visualization was done in UCSF Chimera^65^.

### Fura 2-AM Measurements

The intracellular Ca^2+^ measurements from HEK 293 cells expressing different TRP channel constructs (see above) were performed similarly to those described previously^39,49^. Briefly, to measure 2-APB-dependent inhibition of TRPV6 activity, cells expressing TRPV6 constructs were suspended in Ca^2+^-free buffer and transferred to a quartz cuvette. After an initial 100-s equilibration period, 2-APB was added to the cuvette and incubated for another 100 s, before 2 mM CaCl_2_ was added. For the TRPV6-E402A mutant, 5 mM instead of 2 mM CaCl_2_ was added. For intracellular Ca^2+^ measurements from HEK 293 cells expressing TRPV1, TRPV2, or TRPV3, the experiments were carried out with prewarmed modified HBS (118 mM NaCl, 4.8 mM KCl, 1 mM MgCl_2_, 2.5 mM CaCl_2_, 5 mM d-glucose, 10 mM HEPES pH 7.4). Briefly, HEK 293 cells expressing TRPV1/2/3 channels were centrifuged at 600×*g*, resuspended in modified HBS containing 5 μg/mL of Fura 2-AM (Life Technologies), and incubated at 37 °C for 45 min. Next, the cells were centrifuged for 5 min at 600×*g*, resuspended in prewarmed, modified HBS, and incubated again at 37 °C for 20–30 min in the dark. The cells were subsequently pelleted and washed twice, and then resuspended in modified HBS for fluorescence measurements. Intracellular Ca^2+^ was measured by taking the ratio of fluorescence (510 nm) emitted from Fura 2-AM after excitation at either a wavelength of 340 nm (excites Ca^2+^ bound Fura 2-AM) or 380 nm (excites Ca^2+^ free Fura 2-AM). The excitation wavelength was switched at 1-s intervals. Δ*F*_340_/*F*_380_ was measured as the difference between baseline *F*_340_/*F*_380_ ratio recorded before the addition of Ca^2+^ (for TRPV6 experiments) or 2-APB (for TRPV1-3) and the maximum *F*_340_/*F*_380_ after addition of either Ca^2+^ or 2-APB. Data were normalized to Δ*F*_340_/*F*_380_ in the absence of 2-APB (for TRPV6 constructs) or the Δ*F*_340_/*F*_380_ value at saturating concentrations of 2-APB (for TRPV1/2/3). All Fura2-AM-based fluorescence measurements were conducted using a QuantaMaster^TM^ 40 (Photon Technology International) spectrofluorometer at room temperature in a quartz cuvette under constant stirring.

### Data availability

Data supporting the findings of this manuscript are available from the corresponding author upon reasonable request. Crystal structure coordinates have been deposited in the Protein Data Bank (PDB) under Accession nos. 6D7O (TRPV6*_2-APB_), 6D7P (TRPV6*-Y466A), 6D7Q (TRPV6*-Y466A _2-APB_), 6D7V (TRPV6*_2-APB-Br_), and 6D7X (TRPV6*-Y466A _2-APB-Br_). Cryo-EM density maps have been deposited in the Electron Microscopy Data Bank (EMDB) under Accession nos. EMD-7824 (hTRPV6-Y467A) and EMD-7825 (hTRPV6-Y467A_2-APB_) and the corresponding structure coordinates in the PDB under Accession nos. 6D7S (hTRPV6-Y467A) and 6D7T (hTRPV6-Y467A_2-APB_).

## Electronic supplementary material

Supplementary Information

Description of Additional Supplementary Files

Supplementary Movie 1
