# Supplementary material for: Structural bases of TRP channel TRPV6 allosteric modulation by 2-APB
Source: Nat Commun. 2018 Jun 25;9:2465. doi: 10.1038/s41467-018-04828-y (PMC6018633; doi:10.1038/s41467-018-04828-y)
Supplement: Supplementary file 1 — Supplementary Information [file 41467_2018_4828_MOESM1_ESM.pdf]

Structural bases of TRP channel TRPV6 allosteric modulation by 2-APB

Appu K. Singh[1*], Kei Saotome[1*], Luke L. McGoldrick[1,2] and Alexander I. Sobolevsky[1]

[1]Department of Biochemistry and Molecular Biophysics and [2]Integrated Program in Cellular, Molecular and Biomedical Studies, Columbia University, 650 West 168th Street, New York, NY 10032.

Supplementary Figures 1-3

Supplementary Tables 1-2

Supplementary References

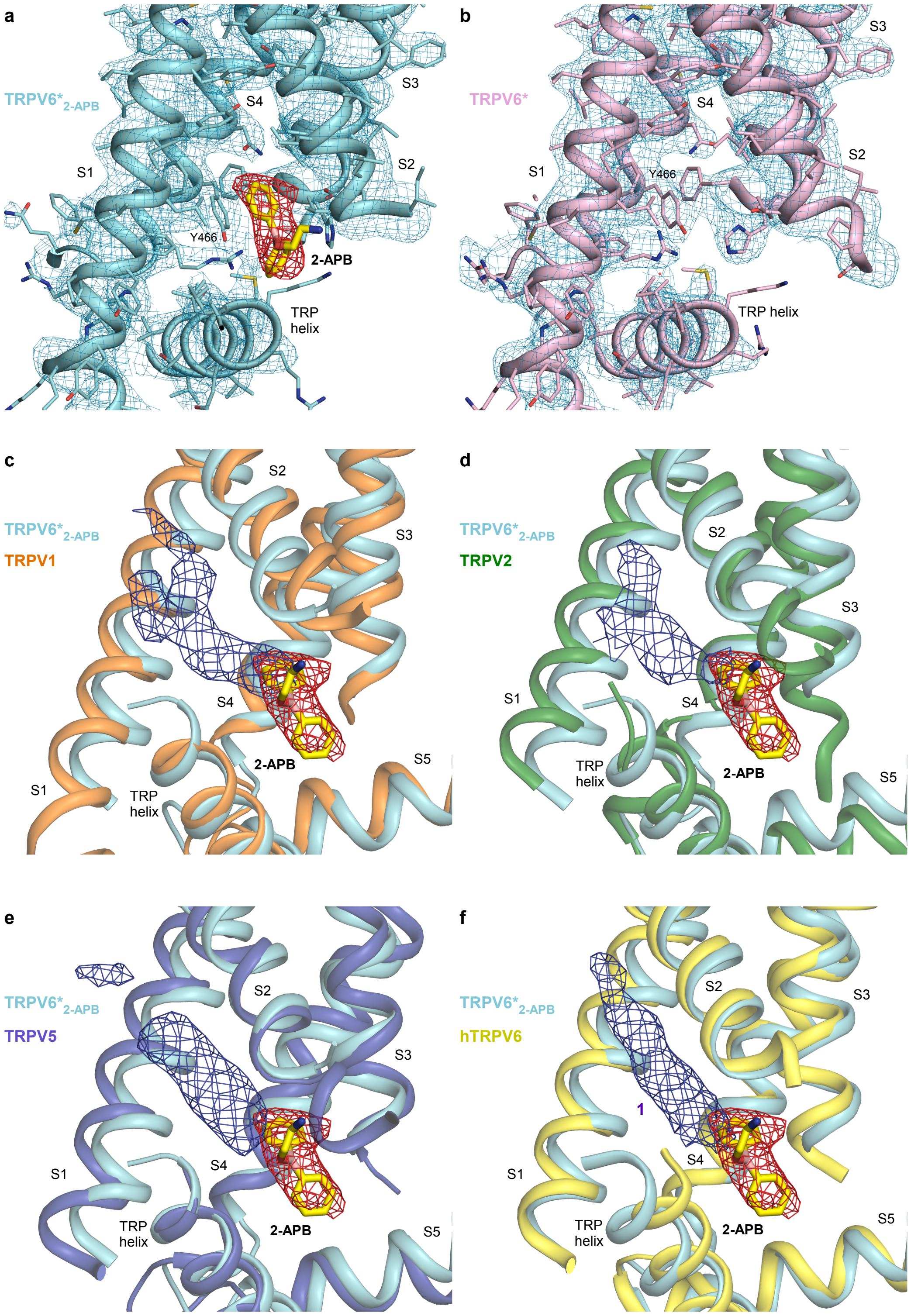

a
TRPV6*2-APB
S1
S2
S3
S4
Y466
2-APB
TRP
helix
b
TRPV6*
S1
S2
S3
S4
Y466
TRP helix
c
TRPV6*2-APB
TRPV1
S1
S2
S3
S4
S5
2-APB
TRP
helix
d
TRPV6*2-APB
TRPV2
S1
S2
S3
S4
S5
2-APB
TRP
helix
e
TRPV6*2-APB
TRPV5
S1
S2
S3
S4
S5
2-APB
TRP
helix
f
TRPV6*2-APB
hTRPV6
1
S1
S2
S3
S4
S5
2-APB
TRP
helix

Supplementary Figure 1. Electron density for 2-APB in the crystal structure of rat TRPV6 and its location compared to the S1-S4 lipids in cryo-EM structures of TRPV channels.

a-b, Close-up view of the 2-APB binding pocket in the crystal structures of TRPV6*$_{2\text{-APB}}$ (**a**) and TRPV6* (**b**) with 2-APB (yellow) and amino acid side chains shown as sticks. Blue mesh shows 2Fo-Fc electron density map contoured at 1σ. Red mesh shows positive electron density for 2-APB in the Fo-Fc omit map contoured at 3σ. **c-f**, Close-up view of superposition of the 2-APB binding pocket in the crystal structure of TRPV6*$_{2\text{-APB}}$ (cyan) and the corresponding regions in the cryo-EM structures of TRPV1 (**c**) PDB ID: 5IRX, orange[1], TRPV2 (**d**) PDB ID: 5AN8, green[2], TRPV5 (**e**) PDB ID: 6B5V, blue[3] and hTRPV6 (**f**) PDB ID: 6BO8 yellow[4]. Purple mesh shows cryo-EM densities for the S1-S4 lipids, interpreted as representations of phosphatidylcholine in TRPV1[1] and TRPV5[3], cholesterol in TRPV2[2] and unknown lipid in hTRPV6[4].

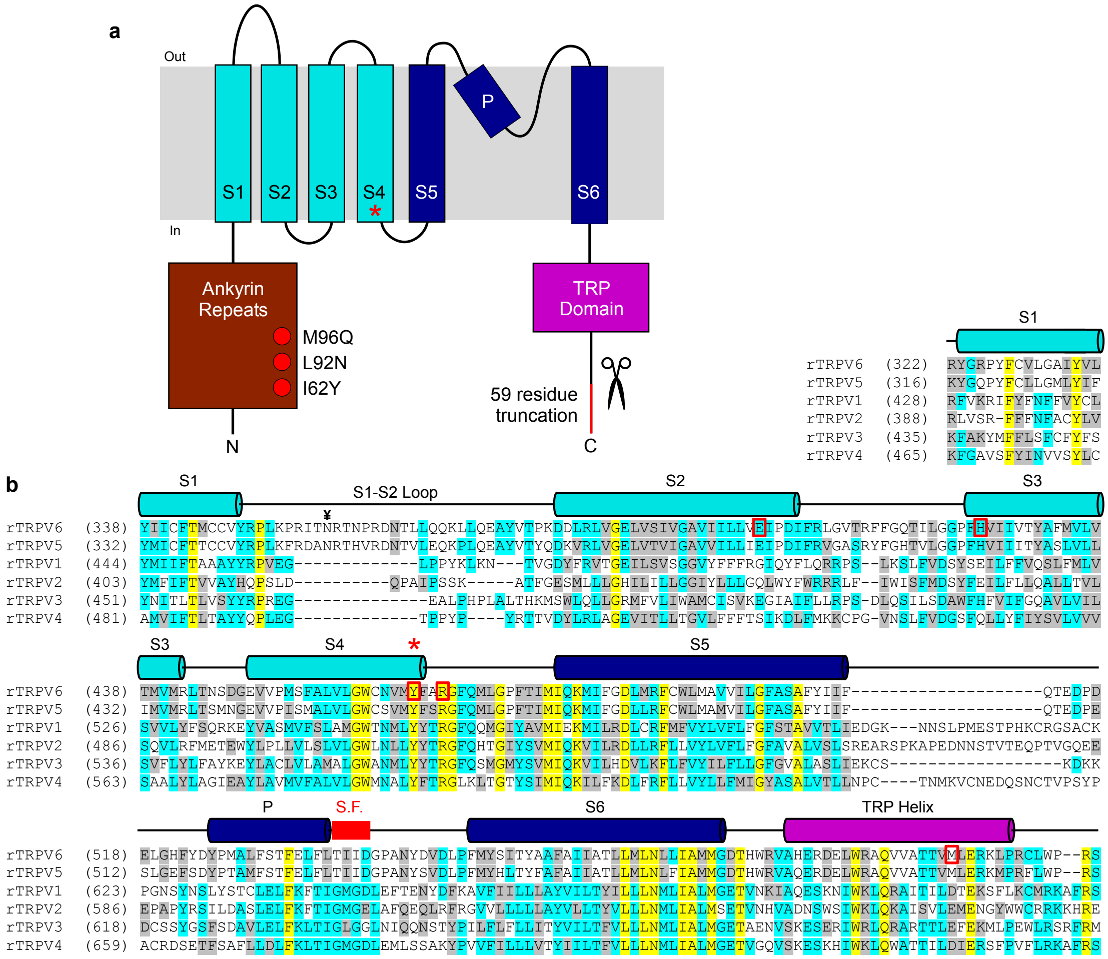
a
Out
In
S1
S2
S3
S4
S5
P
S6
Ankyrin Repeats
M96Q
L92N
I62Y
N
TRP Domain
59 residue truncation
C
S1
rTRPV6 (322) RYGRPYFCVLGAIYVL
rTRPV5 (316) KYGQPYFCLLGMLYIF
rTRPV1 (428) RFVKRIFYFNFFVYCL
rTRPV2 (388) RLVSR-FFFNFACYLV
rTRPV3 (435) KFAKYMFFLSFCFYFS
rTRPV4 (465) KFGAVSFYINVVSYLC
b
S1
S1-S2 Loop
S2
S3
rTRPV6 (338) YIICFTMCCVYRPLKPRITNRTNPRDNTLLQQKLLQEAYVTPKDDLRLVGELVSIVGAVIILLVEIPDIFRLGVTRFFGQTILGGPFHVIIVTYAFMVLV
rTRPV5 (332) YMICFTTCCVYRPLKFRDANRTHVRDNTVLEQKPLQEAYVTYQDKVRLVGELVTVIGAVVILLIEIPDIFRVGASRYFGHTVLGGPFHVIIITYASLVLL
rTRPV1 (444) YMIIFTAAAYYRPVEG-------------LPPYKLKN---TVGDYFRVTGEILSVSGGVYFFFRGIQYFLQRRPS--LKSLFVDSYSEILFFVQSLFMLV
rTRPV2 (403) YMFIFTVVAYHQPSLD----------QPAIPSSK-----ATFGESMLLLGHILILLGGIYLLLGQLWYFWRRRLF--IWISFMDSYFEILFLLQALLTVL
rTRPV3 (451) YNITLTLVSYYRPREG--------------EALPHPLALTHKMSWLQLLGRMFVLIWAMCISVKEGIAIFLLRPS-DLQSILSDAWFHFVIFGQAVLVIL
rTRPV4 (481) AMVIFTLTAYYQPLEG-------------TPPYP----YRTTVDYLRLAGEVITLLTGVLFFFTSIKDLFMKKCPG-VNSLFVDGSFQLLYFIYSVLVVV
S3
S4
S5
rTRPV6 (438) TMVMRLTNSDGEVVPMSFALVLGWCNVMYFARGFQMLGPFTIMIQKMIFGDLMRFCWLMAVVILGFASAFYIIF--------------------QTEDPD
rTRPV5 (432) IMVMRLTSMNGEVVPISMALVLGWCSVMYFSRGFQMLGPFTIMIQKMIFGDLLRFCWLMAMVILGFASAFYIIF--------------------QTEDPE
rTRPV1 (526) SVVLYFSQRKEYVASMVFSLAMGWTNMLYYTRGFQQMGIYAVMIEKMILRDLCRFMFVYLVFLFGFSTAVVTLIEDGK---NNSLPMESTPHKCRGSACK
rTRPV2 (486) SQVLRFMETEWYLPLLVLSLVLGWLNLLYYTRGFQHTGIYSVMIQKVILRDLLRFLLVYLVFLFGFAVALVSLSREARSPKAPEDNNSTVTEQPTVGQEE
rTRPV3 (536) SVFLYLLFAYKEYLACLVLAMALGWANMLYYTRGFQSMGMYSVMIQKVILHDVLKFLFVYILFLLGFGVALASLIEKCS------------------KDKK
rTRPV4 (563) SAALYLAGIEAYLAVMVFALVLGWMNALYFTRGLKLTGTYSIMIQKILFKDLFRFLLVYLLFMIGYASALVTLLNPC----TNMKVCNEDQSNCTVPSYP
P
S.F.
S6
TRP Helix
rTRPV6 (518) ELGHFYDYPMALFSTFELFLTIIDGPANYDVDLPFMYSITYAAFAIIATLLMLNLLIAMMGDTHWRVAHERDELWRAQVVATTVMLERKLPRCLWP--RS
rTRPV5 (512) SLGEFSDYPTAMFSTFELFLTIIDGPANYSVDLPFMYHLTYFAFAIIATLLMLNLFIAMMGDTHWRVAQERDELWRAQVVATTVMLERKMPRFLWP--RS
rTRPV1 (623) PGNSYNSLYSTCLELFKFTIGMGDLEFTENYDFKAVFIILLLAYVILTYILLLNMLIALMGETVNKIAQESKNIWKLQRAITILDTEKSFLKCMRKAFRS
rTRPV2 (586) EPAPYRSILDASLELFKFTIGMGELAFQEQLRFRGVVLLLLLAYVLLTYVLLLNMLIALMSETVNHVADNSWSIWKLQKAISVLEMENGYWWCRRKKHRE
rTRPV3 (618) DCSSYGSFSDAVLELFKLTIGLGGLNIQQNSTYPILFLFLLITYVILTFVLLLNMLIALMGETAENVSKESERIWRLQRARTTLEFEKMLPEWLRSRFRM
rTRPV4 (659) ACRDSETFSAFLLDLFKLTIGMGDLEMLSSAKYPVVFILLLVTYIILTFVLLLNMLIALMGETVGQVSKESKHIWKLQWATTILDIERSFPVFLRKAFRS

Supplementary Figure 2. Topology and sequence alignment of TRPV subunits.

a, Membrane topology of a TRPV6 subunit. Red circles and red line indicate three individual mutations and a C-terminal deletion in the TRPV6* construct, respectively. **b**, Sequence alignment of the transmembrane domain regions in rat TRPV subtypes. Helices are indicated by cylinders above the sequence. Residues lining the 2-APB binding pocket (open red boxes) and the selectivity filter in TRPV6 (thick red line) are highlighted. Red star indicates the highly conserved tyrosine in the 2-APB binding pocket. ¥ marks the N-linked glycosylation site in the extracellular loop connecting S1 and S2 conserved in TRPV6 (and TRPV5) channels.

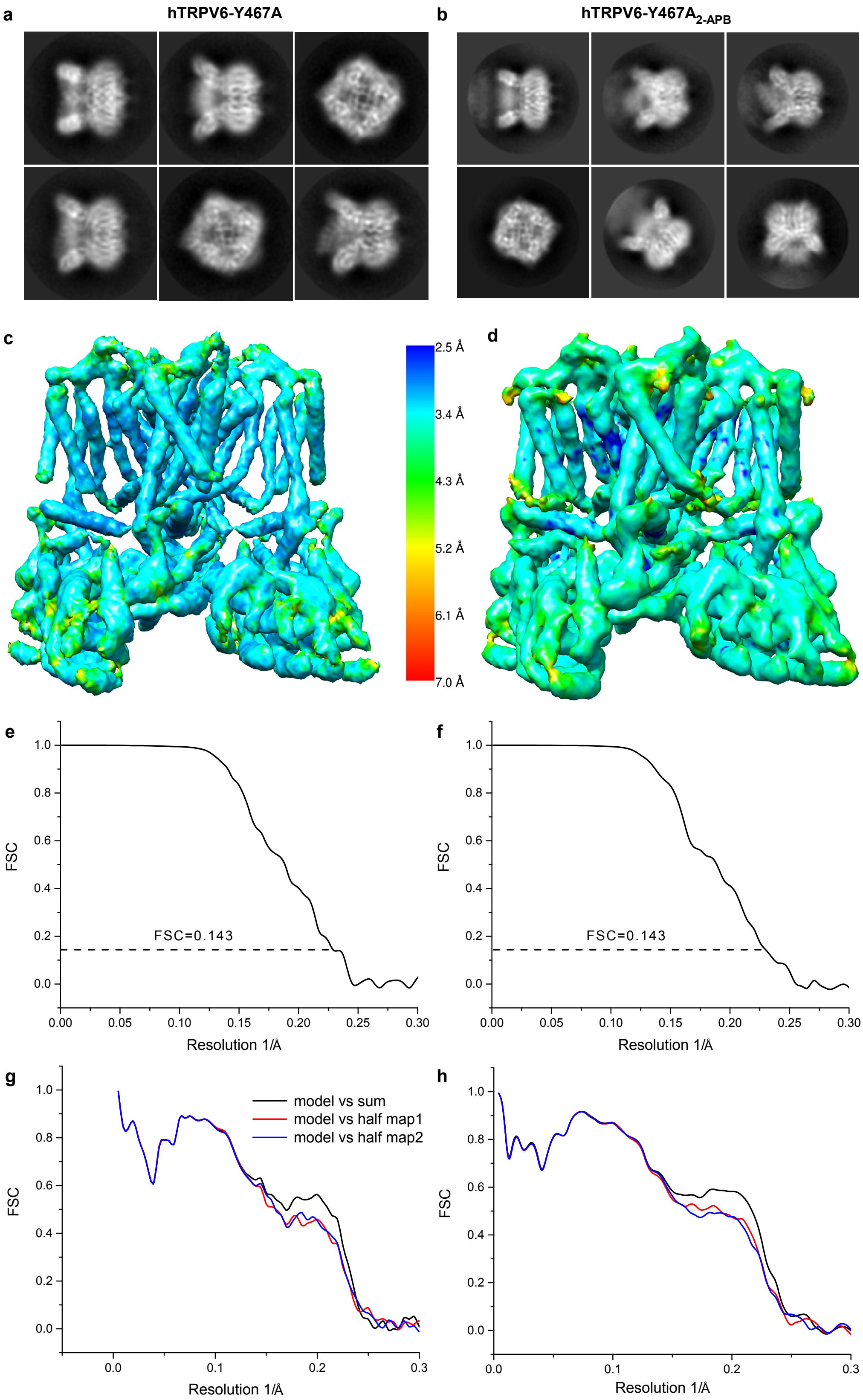

a
hTRPV6-Y467A
b
hTRPV6-Y467A2-APB
c
d
2.5 Å
3.4 Å
4.3 Å
5.2 Å
6.1 Å
7.0 Å
e
f
FSC
FSC=0.143
Resolution 1/Å
g
h
model vs sum
model vs half map1
model vs half map2

Supplementary Figure 3. Overview of single-particle cryo-EM for hTRPV6. a-b, Reference-free two-dimensional class averages of hTRPV6-Y467A (**a**) and hTRPV6-Y467A$_{2\text{-APB}}$ (**b**) illustrating different particle orientations. **c-d**, Local resolution for hTRPV6-Y467A (**c**) and hTRPV6-Y467A$_{2\text{-APB}}$ (**d**) mapped on density calculated using Resmap and two unfiltered half maps, with the highest resolution observed for the channel core. **e-f**, FSC curves for hTRPV6-Y467A (**e**) and hTRPV6-Y467A$_{2\text{-APB}}$ (**f**) calculated between half-maps. **g-h**, Cross-validation FSC curves for hTRPV6-Y467A (**g**) and hTRPV6-Y467A$_{2\text{-APB}}$ (**h**) for the refined model versus unfiltered half maps (only half map1 was used for PHENIX refinement) and the unfiltered summed map.

Supplementary Table 1. Crystallographic data collection and refinement statistics

	TRPV6*$_{2\text{-APB}}$ (PDB ID:6D7O)	TRPV6*-Y466A (PDB ID:6D7P)	TRPV6*-Y466A$_{2\text{-APB}}$ (PDB ID: 6D7Q)	TRPV6*$_{2\text{-APB-Br}}$ (PDB ID: 6D7V)	TRPV6*-Y466A$_{2\text{-APB-Br}}$ (PDB ID: 6D7X)
**Data Collection**					
Beamline	APS-24ID-C	APS-24ID-C	APS-24ID-C	APS-24ID-C	NSLS-II-AMX
Space group	$P42_12$	$P42_12$	$P42_12$	$P42_12$	$P42_12$
Cell dimensions a, b, c, (Å)	145.39 145.39 116.16	146.05 146.05 116.45	145.19 145.19 115.90	144.35 144.35 113.37	144.35 144.35 113.37
α, β, γ (^{0})	90 90 90	90 90 90	90 90 90	90 90 90	90 90 90
Wavelength (Å)	0.9791	0.9791	0.9792	0.92	0.92
Resolution (Å)*	50.00-3.40 (3.62- 3.40)	50.00-3.45 (3.56 - 3.45)	50.00-3.50 (3.92 – 3.50)	50.00–4.50 (4.62- 4.50)	50.00-3.60 (3.69 - 3.60)
Completeness (%)*	94.9 (87.6)	95.8 (96.7)	94.7 (96.3)	99.2 (92.1)	97.7 (98.4)
Redundancy*	8.5 (6.7)	8.7 (8.8)	8.4 (8.5)	16.3 (14.3)	17.0 (10.7)
$\|I/\sigma\|$*	11.44 (1.02)	10.60 (1.08)	7.54 (1.12)	9.10 (1.37)	9.41 (1.33)
R_{meas} (%)**	13.0 (140.9)	14.5 (220.5)	19.4 (250.3)	16.2 (132.3)	19.1 (97.8)
$CC_{1/2}$	99.9 (61.8)	99.9 (52.7)	99.5 (42.5)	99.8 (74.8)	99.6 (71.3)
**Refinement**					
Resolution (Å)*	50.00 - 3.40 (3.62-3.40)	47.21-3.45 (3.66-3.45)	46.90 – 3.50 (3.74 – 3.50)	50.00–4.50 (4.62- 4.50)	50.00 - 3.60 (3.69 - 3.60)
Completeness (%)	94.92 (93.3)	95.76 (96.6)	95.9 (96.1)	94.92 (93.3)	99.8 (98.5)
Number of reflections	16642 (1434)	17374 (1454)	28168 (1558)	16642 (1434)	17758 (791)
R_{work}/R_{free}	0.276/0.281	0.295/0.309	0.293/0.312	0.296/0.301	0.28/0.299
Number of atoms					
Total	4774	4859	4744	4774	4809
Ligand	48	17	46	46	47
B-factor (Å^2)					
Protein	150.47	129.09	146.23	150.47	147.07
2-APB	163.15	NA	103.56	210.59	160.54
CA	62.03	130.87	73.85	NA	NA
DTB	132.38	118.67	119.60	NA	NA
RMS deviations					
Bond length (Å)	0.003	0.003	0.003	0.003	0.003
Bond angles (^{0})	0.55	0.72	0.60	0.55	0.84
Ramachandran					
Favored (%)	94.6	93.5	92.4	94.6	93.6
Allowed (%)	4.9	6.0	6.6	4.9	5.9
Disallowed (%)	0.5	0.5	1.0	0.5	0.5

*Highest resolution shell in parentheses.

5% of reflections were used for calculation of R_{free}.

Supplementary Table 2. Cryo-EM data collection, refinement and validation statistics

	**hTRPV6-Y467A** (EMDB-7824) (PDB ID: 6D7S)	**hTRPV6-Y467A$_{2\text{-}APB}$** (EMDB-*7825*) (PDB ID: 6D7T)
**Data collection and processing**		
Magnification	39,000x	29,000x
Voltage (kV)	300	200
Electron exposure ($e^-/Å^2$)	67	43
Defocus range (μm)	-1.5 to -3.5	-1.5 to -3.5
Pixel size (Å)	0.98	1.22
Symmetry imposed	C4	C4
Initial particle images (no.)	642,142	697,579
Final particle images (no.)	115,126	77,460
Map resolution (Å)	4.34	4.44
FSC threshold		
**Refinement**		
Model resolution (Å)	4.34	4.44
FSC threshold		
Map sharpening B factor ($Å^2$)	-164	-287
Model composition		
Non-hydrogen atoms	19,048	19,040
Protein residues	611	612
Ligands	N/A	4
B factors ($Å^2$)		
Protein	182.9	120.23
2-APB	N/A	148.48
Ca	N/A	145.68
R.m.s. deviations		
Bond lengths (Å)	0.01	0.01
Bond angles (°)	1.08	1.46
Validation		
MolProbity score	1.97	2.69
Clashscore	6.98	9.97
Poor rotamers (%)	1.17	5.48
Ramachandran plot		
Favored (%)	87.78	89.12
Allowed (%)	12.05	10.39
Disallowed (%)	0.17	0.49

Supplementary References

1 Gao, Y., Cao, E., Julius, D. & Cheng, Y. TRPV1 structures in nanodiscs reveal mechanisms of ligand and lipid action. *Nature* **534**, 347-351, doi:10.1038/nature17964 (2016).

2 Zubcevic, L. *et al.* Cryo-electron microscopy structure of the TRPV2 ion channel. *Nature structural & molecular biology* **23**, 180-186, doi:10.1038/nsmb.3159 (2016).

3 Hughes, T. E. T. *et al.* Structural basis of TRPV5 channel inhibition by econazole revealed by cryo-EM. *Nature structural & molecular biology* **25**, 53-60, doi:10.1038/s41594-017-0009-1 (2018).

4 McGoldrick, L. L. *et al.* Opening of the human epithelial calcium channel TRPV6. *Nature* **553**, 233-237, doi:10.1038/nature25182 (2018).
