# Supplementary material for: Structural bases of TRP channel TRPV6 allosteric modulation by 2-APB
Source: Nat Commun. 2018 Jun 25;9:2465. doi: 10.1038/s41467-018-04828-y (PMC6018633; doi:10.1038/s41467-018-04828-y)
Supplement: Supplementary file 2 — Description of Additional Supplementary Files [file 41467_2018_4828_MOESM2_ESM.docx]

**Description of Additional Supplementary Files**

File Name: Supplementary Movie 1

Description:

**Conformational changes in TRPV6 allosterically inhibited by 2-APB.** A morph between open and 2-APB-bound closed states of human TRPV6 represented by the hTRPV6 and hTRPV6-Y467A_2-APB_ structures, respectively. Shown is the entire TRPV6 tetramer and the transmembrane domain of a single subunit viewed parallel to the membrane, and then the entire tetramer and the pore-forming region viewed intracellularly. Sticks represent 2-APB molecules (red), S1-S4 lipids (purple), activating lipids (magenta), residues contributing to the hydrophobic cluster (cyan), residues forming hydrogen bonds (dashed lines) that stabilize the open state (orange) and residues involved in 2-APB binding, and TRPV6 permeation and gating (yellow). The region of S6 that undergoes an α-to-π helical transition during channel opening is colored green.
